# Maladaptive daydreaming should be included as a dissociative disorder in psychiatric manuals: position paper

**DOI:** 10.1192/bjp.2024.279

**Published:** 2025-04

**Authors:** Nirit Soffer-Dudek, Eli Somer, David Spiegel, Richard Chefetz, John O’Neil, Martin J. Dorahy, Etzel Cardeña, Daniel Mamah, Adriano Schimmenti, Alessandro Musetti, Suzette Boon, Annemiek van Dijke, Colin Ross, Ellert Nijenhuis, Annegret Krause-Utz, Paul Dell, Steven N. Gold, Igor Pietkiewicz, Joyanna Silberg, Kathy Steele, Andrew Moskowitz, Nel Draijer, Paula Thomson, Peter Barach, Philip Kinsler, Peter Maves, Vedat Şar, Christa Krüger, Warwick Middleton

**Affiliations:** Department of Psychology, Ben-Gurion University of the Negev, Israel; School of Social Work (Emeritus), University of Haifa, Israel; Psychiatry and Behavioural Sciences, Stanford University, California, USA; The New Washington School of Psychiatry, Washington, DC, USA; McGill University and Teaching Hospital (Emeritus), Montreal, Canada; School of Psychology, Speech & Hearing, University of Canterbury, New Zealand; CERCAP, Department of Psychology, Lund University, Sweden; Department of Psychology, Leiden University, The Netherlands; Department of Psychiatry, Washington School of Medicine, St Louis, Missouri, USA; Department of Human and Social Sciences, Kore University of Enna, Italy; Department of Humanities, Social Sciences and Cultural Industries, University of Parma, Italy; Independent Researcher, private practice, Maarssen, The Netherlands; Leiden University Medical Centre (LUMC), dep National e-health Living Lab (NeLL) and Parnassia/PsyQ Mental Health Institute, The Hague, The Netherlands; The Colin A. Ross Institute for Psychological Trauma, Richardson, Texas, USA; Independent Researcher, Calvos PVL, Portugal; Institute of Psychology, Leiden University, The Netherlands; Churchland Psychological Center, Norfolk, Virginia, USA; Nova Southeastern University (Emeritus), Florida, USA; Research Centre for Trauma and Dissociation, Ignatianum University, Poland; Childhood Recovery Resources, Pikesville, Maryland, USA; Independent Scholar, Atlanta, Georgia, USA; Forensic Psychology Program, The George Washington University, Washington, DC, USA; Department of Psychiatry (Emerita), Vrije Universiteit Amsterdam, The Netherlands; Department of Kinesiology, California State University, California, USA; Case Western Reserve School of Medicine, Cleveland, Ohio, USA; Geisel School of Medicine, New Hampshire, USA; Private Practice, Longmont, Colorado, USA; Department of Psychiatry, Koç University School of Medicine, Turkey; Department of Psychiatry, University of Pretoria, South Africa; Department of Psychology, University of Queensland, Australia

**Keywords:** Dissociative disorders, diagnosis and classification, general adult psychiatry, obsessive–compulsive disorders, trauma and stressor-related disorders

## Abstract

Maladaptive daydreaming is a distinct syndrome in which the main symptom is excessive vivid fantasising that causes clinically significant distress and functional impairment in academic, vocational and social domains. Unlike normal daydreaming, maladaptive daydreaming is persistent, compulsive and detrimental to one’s life. It involves detachment from reality in favour of intense emotional engagement with alternative realities and often includes specific features such as psychomotor stereotypies (e.g. pacing in circles, jumping or shaking one’s hands), mouthing dialogues, facial gestures or enacting fantasy events. Comorbidity is common, but existing disorders do not account for the phenomenology of the symptoms. Whereas non-specific therapy is ineffective, targeted treatment seems promising. Thus, we propose that maladaptive daydreaming be considered a formal syndrome in psychiatric taxonomies, positioned within the dissociative disorders category. Maladaptive daydreaming satisfactorily meets criteria for conceptualisation as a psychiatric syndrome, including reliable discrimination from other disorders and solid interrater agreement. It involves significant dissociative aspects, such as disconnection from perception, behaviour and sense of self, and has some commonalities with but is not subsumed under existing dissociative disorders. Formal recognition of maladaptive daydreaming as a dissociative disorder will encourage awareness of a growing problem and spur theoretical, research and clinical developments.

Maladaptive daydreaming is a distinct syndrome in which the main symptom is excessive vivid fantasising involving immersion in mental imagery of alternative realities that impairs functioning and causes significant distress.^1,2^ Whereas daydreaming is usually a ubiquitous mental activity, often used as a temporary relief, in maladaptive daydreaming it becomes a pathological, chronic and dissociative activity involving persistent engagement in imagined realities, with a detrimental impact on actual life.^1,2^ This frequently exacerbates inattention and detachment from reality, with repeated failed attempts at curbing symptoms. Immersion is often intensified using evocative music and kinaesthetic stereotypies, including facial gestures, circular pacing, whispering of dialogues or physical movements that enact the fantasy content. Although it often provides short-term relief from aversive feelings, such as guilt, shame, low self-worth or loneliness, it also perpetuates these feelings, consequently maintaining psychological suffering. We call for formal recognition of maladaptive daydreaming, suggesting that it be (a) acknowledged as a specific psychiatric disorder in nosographic manuals (e.g. the DSM and ICD) and (b) listed under the dissociative disorders.

## Maladaptive daydreaming as a distinct syndrome

Although pathological immersive fantasising is not a new clinical phenomenon,^3^ maladaptive daydreaming as a distinct concept has garnered recent popularity as individuals actively seek a label for their problem. The development of DSM-like criteria and a structured clinical interview^4^ has enabled gathering of initial epidemiological evidence, resulting in a point-prevalence estimate of 2.5%, with higher rates in younger adults.^5^ Associated distress and dysfunction include prevalent unemployment and suicide attempts.^6,7^ Clinical interview-based research suggests that individuals with maladaptive daydreaming tend to meet criteria for more than one existing disorder, commonly inattention, anxiety, obsessive–compulsive and related disorders, and depression;^8^ however, as we demonstrate below, none of these accounts for its presence or phenomenology. Many patients with maladaptive daydreaming report ineffective treatment when pursuing professional help.^2,9^ Notably, targeted clinical interventions can reduce maladaptive daydreaming and substantially improve well-being;^10^ this indicates the significance of recognising and addressing it.

Accumulated empirical evidence indicates that some individuals temporarily alleviate their distress by immersing themselves in fanciful, imaginative narratives, thereby disrupting their life goals.^6–9,11^ However, rather than representing a disorder, this could be construed as an excessive coping mechanism, providing short-term relief for those with well-established diagnoses (e.g. depression, anxiety). Nevertheless, we argue that there are compelling reasons to justify formalising maladaptive daydreaming as a disorder:

- Maladaptive daydreaming is addictive and structured, substituting other adaptive coping strategies, and additional symptoms are present (e.g. stereotypies, shame, failed attempts to reduce daydreaming, functional impairment), generating a complex clinical picture.
- Maladaptive daydreaming symptoms not only follow psychological distress but also generate and exacerbate it.^6,9^ They tend not to dissipate spontaneously, because maladaptive daydreaming is a stable condition, frequently becoming worse over time. For example, in a 1-year longitudinal study, maladaptive daydreaming was found to be highly stable and prospectively predicted an increase in anxiety and depression symptoms.^12^
- Other DSM or ICD diagnoses do not better explain the clinical picture. Despite significant impaired attention, the mental pattern underlying the distraction – absorptive emotional fantasising – is significantly differentiated from the typical mind-wandering of attention-deficit hyperactivity disorder.^13^ In addition, although maladaptive daydreaming and obsessive–compulsive symptoms are associated,^6,14^ and maladaptive daydreaming could be considered to be compulsive, indulging in a daydream storyline is satisfying and enjoyable in the short term, unlike the distress caused by intrusive obsessions or compulsions. Although some individuals on the autism spectrum have maladaptive daydreaming,^15^ many individuals have maladaptive daydreaming without communication problems and thus do not meet the criteria for autism spectrum disorder. Maladaptive daydreaming could be viewed as a behavioural addiction, but there is no current addiction diagnosis suited for it. Moreover, maladaptive daydreaming does not necessarily include gradually increasing elements to reach the same effect (such as the criterion of increasing money spent in gambling disorder). Maladaptive daydreaming is not characterised by psychotic-spectrum delusions, hallucinations or thought impairments.
- Formally defining maladaptive daydreaming as a psychiatric disorder will increase awareness among clinicians and researchers and facilitate the development of specific intervention protocols.

DSM-like criteria for maladaptive daydreaming have been advanced previously,^4^ but we propose updated criteria (Box 1) based on clinical and research developments of recent years. To avoid over-diagnosis, both the previous and the newly proposed criteria include the standard ‘clinically significant distress or impairment’ criterion of the DSM, ensuring that excessive fantasising is a core source of distress and dysfunction, as well as criteria concerning differential diagnoses and physiological effects. The proposed new criteria are more stringent to accurately pinpoint the unique characteristics of maladaptive daydreaming and avoid over-diagnosis.

Box 1 Proposed diagnostic criteria for maladaptive daydreaming

- Persistent or compulsive vivid and detailed fantasising, characterised by an intense sense of absorption or immersion that includes visual, auditory or affective properties.
- Daydreaming is associated with at least two of the following.
  - A preference for the fantasised experience over real life or a sense of excitement, interest, or emotional attachment to daydreaming that exceeds real-life experiences.
  - Daydreaming is triggered, maintained, or enhanced with stereotypical movement (e.g. pacing, rocking, hand movements), facial gestures, whispering dialogues and/or physical enactments of the storylines.
  - Daydreaming provides short-term relief from aversive feelings such as stress, anxiety, guilt, shame, low self-worth or loneliness.
  - Experiencing annoyance when unable to daydream or when daydreaming is interrupted or curbed.
  - Repeated failed attempts to curb or control daydreaming.
- The symptoms are time-consuming (30 min or more per episode, at least 1 h per day) and have been present for most days in the past 6 months. (A distinction should be made between maladaptive daydreaming and normal mind-wandering, rumination or intrusive thoughts. The latter may also cumulatively take up several hours a day but usually in the context of shorter bouts of spontaneous distraction. In maladaptive daydreaming, specific and significant periods of time are devoted to playing out fantasy scripts, with single episodes reaching 4–5 h in some cases.)
- The symptoms cause clinically significant distress or impairment in social, occupational or other important areas of functioning. Individuals may report that daydreaming provides short-term pleasure but is a core source or maintaining factor for their distress and/or functional impairment.
- The symptoms are not attributable to the physiological effects of a substance or another medical condition (e.g. complex partial seizures).
- The disturbance is not better explained by other disorders such as obsessive–compulsive disorder, attention-deficit hyperactivity disorder, general anxiety disorder, dissociative identity disorder, autism spectrum disorder, post-traumatic stress disorder, acute stress disorder, or major or mild neurocognitive disorder.

Specify severity:

**Mild:** the individual experiences mainly distress, with no apparent functional impairment.

**Moderate:** one area of functioning is affected (e.g. work).

**Severe:** more than one area of functioning is affected (e.g. work, academic or social life).

**Other specified maladaptive daydreaming**: the person meets all criteria for maladaptive daydreaming except for criterion C (duration and/or consumed time).

A mandatory qualification in both sets of criteria is persistent and recurrent fantasy activity characterised by intense absorption or immersion with visual, auditory or affective properties. At least one more characteristic should have been met according to the previous criteria. By contrast, at least two more characteristics are required by the new criteria, and the list is shorter and worded more accurately to capture pathological rather than normal manifestations of daydreaming. As before, it includes stereotypical movement, using daydreaming to cope with distress, feeling annoyed when daydreaming is interrupted, repeated failed attempts to cut back on daydreaming and a preference for daydreaming over real life (e.g. social interactions). The criteria no longer include use of music, boredom, intensification of daydreaming when alone, or preferring daydreaming to daily chores or work, because these happen in the course of ordinary life and are less specific to maladaptive daydreaming. We also added an absolute criterion of time consumed, based on DSM criteria for obsessive–compulsive disorder, specifying at least 1 h per day devoted to immersive fantasising. In addition, a criterion of at least 30 min per daydreaming episode seeks to avoid diagnosing individuals who slip into daydreaming for a few minutes now and then – a normal manifestation of mind-wandering. In maladaptive daydreaming, significant periods of time are specifically devoted to the playing out of fantasy scripts. Individuals who meet all criteria except the duration qualifications may be diagnosed with ‘other specified’ maladaptive daydreaming.

The validity of psychiatric disorders is commonly judged according to five criteria originally set out by Robins and Guze and later Feighner and colleagues^16^; however, well-established psychiatric disorders meet up to three of those criteria. Maladaptive daydreaming meets four criteria: (a) a recognisable clinical presentation, (b) specific and reliable psychological tests for assessment, (c) reliable differentiation from other conditions, and (d) preliminary follow-up validity showing stability.^12^ Research is needed on neurological markers and family studies (see table in the Supplementary material available at https://doi.org/10.1192/bjp.2024.279 for a summary of key studies supporting these assertions). Maladaptive daydreaming research also meets Blashfield’s guidelines for DSM inclusion.^17^ One criterion specifies that the topic be covered by at least 50 published papers in the past decade, including at least 25 empirical studies. Maladaptive daydreaming encompasses much more than that (see the Supplementary material for a list of peer-reviewed papers in the field of maladaptive daydreaming)^a^. Also, in accordance with Blashfield,^17^ diagnostic criteria were developed with solid interrater agreement,^4^ and differentiation was demonstrated. Nonetheless, more research is needed on symptom co-occurrence.

## Maladaptive daydreaming as a dissociative disorder

Although maladaptive daydreaming shares characteristics with various diagnostic categories, including behavioural addictions, obsessive–compulsive disorders, attention-deficit disorders and dissociative disorders,^13,14,18,19^ there are empirical and theoretical reasons for classifying it as a dissociative disorder. Empirically, studies have revealed high rates of maladaptive daydreaming among individuals with severe dissociation and strong associations between maladaptive daydreaming and dissociation.^19,20^ Furthermore, theoretical considerations highlight numerous dissociative characteristics. Individuals with maladaptive daydreaming experience themselves as leading two parallel lives. Internal events dissociate them from external reality, reducing responsiveness to their surroundings and their internal thoughts, feelings, memories, actions and sense of self, resulting in estrangement or disconnection from subjective experience; this is a hallmark of dissociation,^21^ as defined in the DSM-5-TR: ‘Dissociative disorders are characterized by a disruption of and/or discontinuity in the normal integration of consciousness, memory, identity, emotion, perception, body representation, motor control, and behavior’ (p. 330). For example, the typical manifestation of immersion in fantasies while pacing in circles in one’s room represents a disconnect between perception and behaviour on one hand and consciousness and attention on the other. Maladaptive daydreaming may lead to experiential disconnectedness and self-incoherence,^21^ with individuals exploring different personas and characters in a complex inner world. Characters are imagined as having distinct personalities, desires and conflicts. The scripts in one’s fantasy world may be experienced with varying degrees of control and agency. This increases absorption in the daydream and disconnects from other perceptions, memories and sense of self, producing momentary relief and pleasure. An experience of playing out an ideal fantasy self in one’s mind is commonly reported to be at odds with one’s real-life self, leading to a discontinuity in one’s sense of identity.

Maladaptive daydreaming is both similar to and different from current dissociative disorders. Regarding depersonalisation/derealisation disorder (DP/DRD), vivid fantasisers often feel that their daydreams are more real than actual life, resulting in depersonalisation–derealisation or a detached sense of reality when not daydreaming.^19^ In a daily diary study, increased depersonalisation–derealisation experiences were reported on days of increased daydreaming and the following day but not the preceding day,^6^ supporting the directionality mentioned above. Both DP/DRD and maladaptive daydreaming may or may not follow trauma but are significantly associated with difficulties in regulating emotion (e.g.^22–25^). However, a DP/DRD diagnosis does not necessarily imply daydreaming.

Regarding dissociative amnesia, individuals with maladaptive daydreaming are often lost in daydreams for several hours, and, when they ‘snap out of it’, they may experience a sense of lost time. However, they retain accessible memories of the fantasies they experienced and their autobiographical information. Regarding dissociative identity disorder (DID), both maladaptive daydreaming and DID imply mental and emotional engagement with different personas and characters with distinct personalities, desires and conflicts in a complex inner world. However, maladaptive daydreaming involves no division of personality or switching *per se*. Maladaptive daydreaming dramas occur in fantasy and are not acted out in the real world. This difference also applies to ‘subclinical’ DID, either the DSM’s ‘other specified dissociative disorder, example 1’, or the ICD’s partial DID. Finally, using the ‘unspecified’ or ‘other specified’ categories to diagnose maladaptive daydreaming is not ideal, as these are meant for rare or atypical presentations. By contrast, maladaptive daydreaming is a prevalent, specific, prototypical set of presenting symptoms. Its addition as a stand-alone disorder would enable accumulation of scientific and clinical knowledge on aetiology, phenomenology and efficient treatment courses.

Generally, the proposed classification of maladaptive daydreaming as a dissociative disorder aligns with its phenomenological characteristics, including ‘double consciousness’^19^ in the simultaneous experience of reality and fantasy, partial (phenomenologically experienced) dissociative intrusions,^26^ and concomitant disconnection from ordinary self-awareness and external perception. Therefore, including maladaptive daydreaming in the dissociative disorders sections of psychiatric manuals could advance research on shared mechanisms, such as alterations in the sense of agency, imagination, suggestibility, self-coherence and automaticity; thus, it could contribute to our understanding of a range of dissociative processes, including compartmentalisation of emotions, multiplicity, memory errors, disruptions in phenomenological and narrative self, differences between dissociated and non-dissociated psychological states, and complex DID presentations.

## Additional considerations, limitations and a need for further research

The aetiological factors determining maladaptive daydreaming are not yet firmly established; however, several factors have been hypothesised to play a part in the development of this condition. First, it has been hypothesised that an innate ability for immersive daydreaming, which may not in itself be psychopathological, constitutes a risk factor or diathesis for maladaptive daydreaming.^19^ Reports of extensive immersive daydreaming starting at very early ages are the norm,^9^ whereas the problematic aspect of it may develop over time. Relatedly, some young children diagnosed with stereotypical movement disorder experience extensive imagery accompanying the stereotypies, and this may evolve into adult maladaptive daydreaming.^27^ Genetic factors may play a part in these developmental tendencies, but this has not yet been empirically researched. Second, similarly to dissociation, stress or trauma may have a vital role, facilitating the metamorphosis of the heightened imagery tendency into an impairing psychopathology. Specifically, maladaptive daydreaming is often associated with past trauma (e.g.^23–24,28^, but see^9,11^) or with a current aversive or stressful reality such as strenuous relationships at home^19^ or social anxiety.^28^ Shame and secrecy may also have roles in both maladaptive daydreaming and dissociation.^21^ In addition, several studies have shown that maladaptive daydreaming is associated with difficulties in emotion regulation (e.g.^25^), which could play an aetiological part by strengthening the need for an alternative coping mechanism. Finally, personality dynamics also contribute to this condition. Specifically, there seems to be a central connection between maladaptive daydreaming and narcissism, with a majority of a clinical sample with narcissistic personality disorder exhibiting probable maladaptive daydreaming.^29^

Some limitations of the current work should be acknowledged. First, despite the apparent evidence supporting the existence of maladaptive daydreaming as a clinical phenomenon, more research is needed to establish clearly the boundaries between normal and pathological daydreaming. In particular, the field will benefit most from neuropsychological research pinpointing the brain mechanisms involved, longitudinal research on children and teens to shed light on the typical developmental trajectory, and experimental work uncovering process dynamics in the maintenance of the condition. In addition, the diagnostic criteria suggested in this work, although based on clinical and research experience accumulated in recent years, are still somewhat arbitrary; further research is needed to quantify their ability to differentiate clinical from subclinical presentations. Importantly, interrater agreement rates exist for the previous criteria, but rates for the new criteria will also be required to determine adequate validity. Follow-up validity requires longitudinal research on interview-diagnosed individuals.

There is also concern that adding maladaptive daydreaming to psychiatric manuals would contribute to the general problem of increasing the number of diagnoses in those manuals. Notably, however, fugue as a separate disorder was omitted in the latest version of the DSM, so that may be less of a problem in the field of dissociative disorders. Finally, the dissociative nosology suggested here is not the only possible way to conceptualise maladaptive daydreaming. Despite its significant dissociative components, it could arguably be categorised as a behavioural addiction, an obsessive–compulsive spectrum disorder or a unique subtype of attention-deficit hyperactivity disorder. Indeed, some psychiatric disorders are hard to classify, even though they have distinct patterns and their definition is clinically useful,^30^ as they may have characteristics that overlap with more than one taxonomy dimension. The present position paper is a statement from a significant proportion of leaders in the field of dissociation who see the relevance and utility of adding maladaptive daydreaming to the spectrum of dissociative disorders.

To conclude, given that maladaptive daydreaming has substantial empirical support, a distinct clinical presentation and a significant impact on individuals’ lives, it warrants recognition as a dissociative disorder in diagnostic manuals. This would acknowledge the validity of maladaptive daydreaming, guide clinicians in accurate diagnosis, stimulate further research, and ensure that affected individuals who have long felt overlooked receive appropriate support and effective treatments.

## Supporting information

Soffer-Dudek et al. supplementary material Soffer-Dudek et al. supplementary material

## Data Availability

Data availability does not apply to this article, as no new data were created or analysed in this study.

## Footnotes

a For a list that is continuously updated please visit: https://daydreamresearch.wixsite.com/md-research/publications.
