# Supplementary material for: Maladaptive daydreaming should be included as a dissociative disorder in psychiatric manuals: position paper
Source: Br J Psychiatry. 2025 Mar 17;226(4):238–42. doi: 10.1192/bjp.2024.279 (PMC12038384; doi:10.1192/bjp.2024.279)
Supplement: Soffer-Dudek et al. supplementary material [file S0007125024002794sup001.docx]

**Supplementary materials file for the article:**

**“Maladaptive Daydreaming Should Be Included as a Dissociative Disorder in Psychiatric Manuals: A Position Paper.”**

Table S1

A summary of some of the central studies supporting the validity of maladaptive daydreaming as a disorder, according to Robins and Guze criteria.

| **Robins and Guze criteria** | **Supporting studies** | **Need for further research** |
| --- | --- | --- |
| A recognizable clinical presentation | A clear prototypical clinical presentation emerged from several studies including case studies, qualitative explorations, and quantitative research, both cross-sectional and daily diary studies. For example:  Bigelsen & Schupak (2011)  Bigelsen et al. (2016)  Chaudhary et al. (2022)  Margherita et al. (2022)  Nowacki & Pyszkowska (2024)  Pietkiewicz et al. (2018)  Pietkiewicz et al. (2023)  Schimmenti et al. (2019a)  Schupak & Rosenthal (2009)  Sharma & Mahapatra (2021)  Soffer-Dudek & Somer (2018)  Somer (2002)  Somer (2018)  Somer (2023)  Somer et al. (2016a,b)  Somer et al. (2019)  Wen et al. (2022) |  |
| Specific and reliable psychological tests | The MDS-14 self-report scale was validated in Somer et al. (2016c)  The MDS-16 is a later development in which two items were added (Somer et al., 2017a); it was translated and validated in several languages, such as Italian (Schimmenti et al., 2019b), Hungarian (Sándor et al., 2020), Polish (Pietkiewicz et al., 2023), Ukranian (Balashevich et al., 2024), Brazilian Portuguese (Catelan et al., 2023), Arabic (Abu-Rayya et al., 2019), Turkish (Metin et al., 2022), Persian (Ahmadi et al., 2022), and Hebrew (Jopp et al., 2018). A large-scale cross-cultural research revealed its measurement invariance, supporting reliability and validity for most items (Soffer-Dudek et al., 2020)  The MDS-SF5 is a recent development comprising 5 central items, shown to discriminate similarly to the full MDS (Soffer-Dudek & Oh, 2024)  The SCIMD is a structured clinical interview based on the previous diagnostic criteria, that showed excellent inter-rater agreement (Somer et al., 2017b) | Neurological markers not yet available |
| Reliable differentiation from other conditions | A structured clinical interview study showed significant co-morbidity but also distinction by establishing co-morbidity rates between maladaptive daydreaming and additional diagnoses such as depression, anxiety, and obsessive-compulsive disorder (Somer et al., 2017a)  A structured clinical interview study supported a distinction between maladaptive daydreaming and attention-deficit/hyperactivity disorder (Theodor-Katz et al., 2022)  A clinical interview large-scale study showed significant comorbidity, but also distinction, of maladaptive daydreaming from autism spectrum disorder (West et al., 2022)  A study based on self-report clinical cutoffs found significant co-morbidity but also significant distinction between maladaptive daydreaming and obsessive-compulsive disorder (Salomon-Small et al., 2021) |  |
| Follow-up validity showing stability | Preliminary support for construct stability from a 1-year longitudinal study with a large sample (Musetti et al., 2023) | Need for longitudinal research on interview-diagnosed individuals |
| Family studies |  | No data available yet on family prevalence rates |

**List S1: Published papers on maladaptive daydreaming**

**The following list includes most or all accepted or published peer-reviewed articles or chapters where “Maladaptive Daydreaming” or otherwise labeled pathological daydreaming is the primary topic or one of the primary topics. We cannot guarantee that this list is exhaustive.**

**The following list was updated in October 2024. For a list that is continuously updated please visit: https://daydreamresearch.wixsite.com/md-research/publications.**

2024

141. Mancinelli, E., Spisto, S., Sukhija, V.J. & Salcuni, S. (2024). Maladaptive daydreaming as emotion regulation strategy: exploring the association with emotion regulation, psychological symptoms, and negative problem-solving orientation. Current Psychology. https://doi.org/10.1007/s12144-024-06487-3

140. Soffer-Dudek, N. (2024). Seeing is believing: implications of the dreamlike cognitive style for waking spontaneous thought and psychopathology. Philosophy and the Mind Sciences, 5. https://doi.org/10.33735/phimisci.2024.10208

139. Nowacki, A. & Pyszkowska, A. (2024). The everchanging maladaptive daydreaming – a thematic analysis of lived experiences of Reddit users. Current Psychology. https://doi.org/10.1007/s12144-024-06506-3

138. Nowacki, A. & Pyszkowska, A. (2024). It is all about discomfort avoidance: maladaptive daydreaming, frustration intolerance, and coping strategies – a network analysis. Current Psychology. https://doi.org/10.1007/s12144-024-06382-x

137. Jain, S. (2024). Maladaptive and Negative Maladaptive Daydreaming Among Youth & Impact of Emotional Neglect on MD. International Journal of Indian Psychology, 12(2). https://doi.org/10.25215/1202.194

136. Öğü, Ç (2024). Association of maladaptive daydreaming with behavioral addiction, Health Sciences Quarterly 4(3), 233-241. https://journals.gen.tr/jsp.

135. Pezzi, M., Zagaria, A., Miguel-Alvaro, A., Gámez-Guadix, M., Gori, A., Santoro, G., & Musetti, A. (2024). Maladaptive Daydreaming and Problematic Online Behaviors: A Network Analysis Approach. Journal of Psychiatric Research. https://doi.org/10.1016/j.jpsychires.2024.07.023

134. Shimoni, H., & Axelrod, V. (2024). Elucidating the difference between mind-wandering and day-dreaming terms. Scientific Reports, 14(1), 11598. https://doi.org/10.1038/s41598-024-62383-7

133. Chávez, J.E. (2024). La ensoñación excesiva como una respuesta al estrés en adolescentes (Maladaptive daydreaming as a stress response in adolescents). Educación y Salud - Boletín Científico Instituto de Ciencias de la Salud Universidad Autónoma del Estad, 12 (24), 47-52. https://doi.org/10.29057/icsa.v12i24.12754

132. Roy, A., Girija, V. S., & Kitzlerová, E. (2024). The Role of Momentary Dissociation in the Sensory Cortex: A Neurophysiological Review and its Implications for Maladaptive Daydreaming. Medical Science Monitor: International Medical Journal of Experimental and Clinical Research, 30, e944209. https://doi.org/10.12659/MSM.944209

131. Saladino, V., Calaresi, D., Cuzzocrea, F., and Verrastro, V. (2024). The Interplay between Binge Watching and Suicide Risk: Daytime Sleepiness and Maladaptive Daydreaming as Mediators. Social Sciences, 13, 283. https://doi.org/10.3390/ socsci13060283

130. Zorlu, Ö., Beyazũz, E., Aytekin, S. & Albayrak, H. (2024). Maladaptive Daydreaming in Psoriasis Patients. Namık Kemal Medical Journal, 12(2), 85-92. https://doi.org/10.4274/nkmj.galenos.2024.26349.

129. Constantine, L. & Haque, S. (2024). Loneliness, Self-Esteem, and Maladaptive Daydreaming in University Students: The Mediating Role of Perceived Stress. Research Square. https://doi.org/10.21203/rs.3.rs-4538726/v1

128. Balashevich, O.K., Harahukiua, A.I, Molotokas, A.A., Bayer, O.O, Kurapov, A.O. (2024). Adaptation and Psychometric Evaluation of the Maladaptive Daydreaming Scale (MDS-16) on a Ukrainian Sample: A Pilot Study. Perspectives and Innovations of Science, 6(40), 609-629. https://doi.org/10.52058/2786-4952-2024-6(40)-609-629

127. Hedderly, T., Eccles, C., Malik, O., Abdulsatar, F., Mitchell, C., Owen, T., Soffer-Dudek, N., Grose, C., Fernandez, T.V., Robinson, S. and Somer, E. (2024). Intense Imagery Movements May Lead to Maladaptive Daydreaming: A Case Series and Literature Review. Movement Disorders Clinical Practice, 11, 716-719. https://doi.org/10.1002/mdc3.14011

126. Saladino V, Calaresi D, Cuzzocrea F, Verrastro V. (2024). The Interplay between Binge Watching and Suicide Risk: Daytime Sleepiness and Maladaptive Daydreaming as Mediators. Social Sciences, 13(6):283. https://doi.org/10.3390/socsci13060283

125. Somer, E. & Otgaar, H. (2024). Exploring the Nexus between Childhood Adversities, Trauma-related Fantasy and Memory in Maladaptive Daydreaming. Acta Psychologica, 247, 104301.  https://doi.org/10.1016/j.actpsy.2024.104301

124. Aruguete, M.S., Grieve, F., Zsila, Á. et al. The absorption-addiction model of celebrity worship: in search of a broader theoretical foundation. BMC Psychology 12, 224 (2024). https://doi.org/10.1186/s40359-024-01733-6

123. Shiota, S., Oura, S.I., & Matsumoto M. (2024). Maladaptive fantasy predicts negatively distorted self and other mental representation: A consideration of child abuse from psycho/neuro/biological perspectives. Environment and Social Psychology, 9(7): 2079. https://doi.org/10.59429/esp.v9i7.2079

122. Begum, A. & Khan, M.J. (2024). Childhood Emotional Maltreatment and Maladaptive Daydreaming Among Adolescents: Mediating Role of Emotional Processing. Pakistan Journal of Psychological Research, 39 (1) 123-137. https://doi.org/10.33824/PJPR.2024.39.1.08

121. Somer, E. (2024). When Imagination Feels Like Reality: A Case Study of False Memories and Maladaptive Daydreaming in Visual Impairment. Case Reports in Psychiatry, Article ID 9391645, 82024. https://doi.org/10.1155/2024/9391645.

120. Lawson, E. & Thompson, E. (2024). Daydreaming as spontaneous immersive imagination: A phenomenological analysis. Philosophy and the Mind Sciences, 5. https://doi.org/10.33735/phimisci.2024.9913

119. Balashevych, O.K., & Molotokas, A.A. (2024). Comparative Analysis of Maladaptive Daydreaming and Other Forms of Imagination. Habitus. https://doi.org/10.32782/2663-5208.2024.57.56 (In Ukrainian).

118. Shanbhag, T., & Pothiyil, D.I. (2024). A Cognitive Approach to Maladaptive Daydreaming: A Case Report. Indian Journal of Psychological Medicine. doi:10.1177/02537176241236898

117. Somer, E. (2024). Calling the tune in maladaptive daydreaming: The impact of music on the experience of compulsive fantasizing. Psychology of Music. https://doi.org/10.1177/03057356231222290

116. Soffer-Dudek, N., & Oh, H. (2024). Maladaptive daydreaming: A shortened assessment measure and its mental health correlates in a large Unites States sample. Comprehensive Psychiatry, 129, 152441. https://doi.org/10.1016/j.comppsych.2023.152441

2023

115. Soffer-Dudek, N. (2023). Obsessive-compulsive symptoms and dissociative experiences: suggested underlying mechanisms and implications for science and practice. Frontiers in Psychology, 14, 1132800. https://doi.org/10.3389/fpsyg.2023.1132800

114. Sharma, M. & Jain, V. (2023). Fear of COVID-19 in Relation to Social Connectedness, Maladaptive Daydreaming, Depression and Anxiety: A Correlation Study. Indian Journal of Health and Well-being, 14(4), 491-494.

113. Catelan, R.F.,Zsila, Á., Pietkiewicz, I.J., & Nardi, A.E. (2023). Cross-cultural adaptation and validation of the Brazilian Portuguese version of the Maladaptive Daydreaming Scale (BMDS-16). Psychiatria I Psychologia Kliniczna, 23 (3), 129–140. https://dx.doi.org/10.15557/PiPK.2023.0018.

112. Jahangiri, N., Hashemi, N., Jafari, L., Abdollahi Siyahkaldeh, F. (2023). Prediction of Psychological Distress and Job Performance of Nurses Based on Maladaptive Daydreaming and Social Loneliness during the Covid 19 Epidemic. Quarterly Journal of Nursing Management, 11(4), 37-47 مدیریت پرستاری. URL: http://ijnv.ir/article-1-978-en.html

111. Pyszkowska, A., Celban, J., Nowacki, A., & Dubiel, I. (2023). Maladaptive daydreaming, emotional dysregulation, affect and internalized stigma in persons with borderline personality disorder and depression disorder: A network analysis. Clinical Psychology & Psychotherapy. https://doi.org/10.1002/cpp.2923

110. Chefetz, R. A., Soffer-Dudek, N., & Somer, E. (2023). When daydreaming becomes maladaptive: phenomenological and psychoanalytic perspectives. Psychoanalytic Psychotherapy, 37:4, 319-338, DOI: 10.1080/02668734.2023.2246058

109. Somer, E. (2023). Body Movements During Maladaptive Daydreaming: A Thematic Analysis of Asynchronous Email Interviews. Journal of Anomalous Experience and Cognition, 3(1), 267-299. https://doi.org/10.31156/jaex.25004.

108. Vyas, M., Shaikh, M., Rana, S. & Pendyala, A. (2023). Is this the real life? Or just a fantasy? A closer look at maladaptive daydreaming. Mental Health and Social Inclusion. https://doi.org/10.1108/MHSI-01-2023-0014.

107. Lakshyay Rawat, A. & Malik, H. (2023). Daydreaming: Vex or Delight? International Journal of Engineering Technology and Management Sciences 3(7), 105. https://doi.org/10.46647/ijetms.2023.v07i03.105 (pdf).

106. Thomson P. & Victoria Jaque, S. (2023): Maladaptive Daydreaming, Overexcitability, and Emotion Regulation, Roeper Review, 45(3), 195-205. https://doi.org/10.1080/02783193.2023.2212634

105. Thomson, P. & Victoria Jaque, S. (2023). Creativity, Emotion Regulation, and Maladaptive Daydreaming. Creativity Research Journal, 1-10. https://doi.org/10.1080/10400419.2023.2230022

104. Nauman, J., (2023). Narrative Review of a Link Between Proposed Maladaptive Daydreaming (MD) and Reports of Motor Stereotypy in Autism Spectrum Disorder. Review Journal of Autism and Developmental Disorders, 1-7. https://doi.org/10.1007/s40489-023-00386-2

103. Chauhan , N., Sharma, N., & Mahajan, S. (2023). Beguiling daydreams: a case of maladaptive daydreaming. Prime Care Companion for CNS Disorders, 25(3), 47372. https://doi.org/10.4088/PCC.22cr03355

102. Pietkiewicz, I.J., Hełka, A.M., Barłóg, M., & Tomalski, R. (2023). Maladaptive daydreaming and narcissism. Personality and Individual Differences, 212, 112279. https://doi.org/10.1016/j.paid.2023.112279.

101. Mishra, B. & Kewalramani, S. (2023). Social Media Use, Maladaptive Daydreaming, and Imposter Phenomenon in Younger Adults. Journal of Advance Research in Science and Social Science, 6(1). https://doi.org/10.46523/jarssc.06.01.20

100. Shafiq, S., Zafar H., & Khalid, N. (2023). Development and validation of dysfunctional daydreaming scale. Journal of Postgraduate Medical Institute, 37(1), 21-26. http://doi.org/10.54079/ jpmi.37.1.3087

99. Musetti, A., Soffer-Dudek, N., Imperato, C., Schimmenti, A., & Franceschini, C. (2023). Longitudinal associations between maladaptive daydreaming and psychological distress during the COVID-19 health crisis. Journal of Behavioral Addictions, 12(1), 288-294. https://doi.org/10.1556/2006.2023.00001

98. Pietkiewicz, I. J., Hełka, A., Barłóg, M., & Tomalski, R. (2023). Validity and reliability of the Polish Maladaptive Daydreaming Scale (PMDS-16) and its short form (PMDS-5). Clinical Psychology & Psychotherapy. 30(4), 882–897. https://doi.org/10.1002/cpp.2844

97. Ghinassi, S., Fioravanti, G., & Casale, S. (2023). Is shame responsible for maladaptive daydreaming among grandiose and vulnerable narcissists? A general population study. Personality and Individual Differences, 206, 112122. https://doi.org/10.1016/j.paid.2023.112122

96. Jahangiri, N., Hashemi, N., jafari, L., & Abdollahi Siyahkaldeh, F. (2023). Prediction of Psychological Distress and Job Performance of Nurses Based on Maladaptive Daydreaming and Social Loneliness during the Covid-19 Epidemic. Quarterly Journal of Nursing Management, 11(4), 37-47. (in Farsi). (English Abstract).

95. Herscu, O., Somer, E., Federman, A., & Soffer-Dudek, N. (2023). Mindfulness meditation and self-monitoring reduced maladaptive daydreaming symptoms: A randomized controlled trial of a brief self-guided web-based program. Journal of Consulting and Clinical Psychology, 91(5), 285-300. https://doi.org/10.1037/ccp0000790

94. West, M. J., Somer, E., & Eigsti, I. M. (2023). Immersive and maladaptive daydreaming and divergent thinking in autism spectrum disorders. Imagination, cognition and personality, 42(4), 372-398. https://doi.org/10.1177/02762366221129819

93. Anandarami, V.S. & Roneena, A.J. (2023). Maladaptive daydreaming in individuals with disorganized attachment style: A case study. Journal of Research in Social Sciences and Humanities, 3(2). https://doi.org/10.47679/jrssh.v3i2.42

2022

92. Roneena, A.J. & Anandarani, V.S. (2022). Mindfulness-Based Cognitive Therapy (MBCT) for Maladaptive daydreaming: A Case Report. International Research Journal of Education and Technology, 4(10).

91. Horváth-Labancz, E., Sándor, A., Balázs, K., Molnár, J., & Kuritárné Szabó, I. (2022). Pathological personality traits of maladaptive daydreamers measured by the Personality Inventory for DSM-5 in a psychiatric sample. Clinical psychology & psychotherapy, 30(3), 536-547. https://doi.org/10.1002/cpp.2820

90. Kamak Sürman, Z. (2022). Uyum Bozucu Gündüz Düşleri : Psikiyatri Etiği Açısından Yaklaşım. (Ethical Approach to Maladaptive Daydreaming) Türkiye Biyoetik Dergisi, (Turkish Journal of Bioethics) 9(3), 120-122. DOI: 10.5505/tjob.2022.21033

89. Ahmadi, F., Goodarzi, M., Kazemi Rezai, S.A. & Yazdanimehr, R. (2022). Reliability, validity, and factor structure of the maladaptive daydreaming scale (MDS-16) in an Iranian sample. Journal of Clinical Psychology, 14(4), 53-63. DOI: 10.22075/JCP.2022.27056.2443

88. Rana, S. & Vyas, (2022). Maladaptive daydreaming: Overview. International Journal of Social Sciences, 10(3), 370-375.

87. Jain, S. (2022). Maladaptive and Negative Maladaptive Daydreaming Among Youth & Impact of Emotional Neglect on MD. International Journal of Indian Psychology, 12(2). https://doi.org/10.25215/1202.194

86. Seth, M. & Bhargava, K. (2022). Maladaptive Daydreaming as A Coping Mechanism to Escape Loneliness. International Journal of Indian Psychology, 12(2). https://doi.org/10.25215/1202.163

85. Chaudhary, S., Jain, K., Agarwal, M. & Bajaj, V. (2022). Maladaptive Daydreaming Among the Indian Youth: A Qualitative and Quantitative Analysis. International Journal of Indian Psychology, 10(1). https://doi.org/10.25215/1001.027

84. Shafiq, S. & Zafar, H. (2022). Social Anxiety as Predictor of Depression in Adolescents: Mediating Role of Dysfunctional Daydreaming. Journal of Liaquat University of Medical and Health Sciences, 21(04), 301-305. doi.10.22442/jlumhs.2022.00963

83. Soffer-Dudek, N. & Somer, E. (2022). Maladaptive daydreaming is a dissociative disorder: Supporting evidence and theory. In J.M. Dorahy (ed.) Dissociation and the Dissociative Disorders: Past, present, future (2nd ed.). Taylor & Francis, pp 547-559.

82. Chirico, I., Volpato, E., Landi, G. et al. (2022). Maladaptive Daydreaming and Its Relationship with Psychopathological Symptoms, Emotion Regulation, and Problematic Social Networking Sites Use: a Network Analysis Approach. International Journal of Mental Health and Addiction. 1-17. https://doi.org/10.1007/s11469-022-00938-3

81. West, M.J., Somer, E., & Eigsti, I.-M. (2022). Shared Challenges and Cooccurrence of Maladaptive Daydreaming and Autism Spectrum Disorder. Advances in Neurodevelopmental Disorders, 7(1), 77-87. https://doi.org/10.1007/s41252-022-00279-1

80. Musetti, A., Gori, A., Michelini, G., Di Monte, C., Franceschini, C., & Mariani, R. (2022). Are defense styles mediators between traumatic experiences and maladaptive daydreaming? Current Psychology, 42(30), 26683-26691. https://doi.org/10.1007/s12144-022-03708-5

79. Thorburn, C. (2022). ‘Maladaptive Daydreaming’: An introduction to a new condition. European Psychiatry, 65(S1), S178-S179. https://doi.org/10.1192/j.eurpsy.2022.473

78. Wen, H., Soffer-Dudek, N., & Somer, E. (2022). Daily feelings and the affective valence of daydreams in maladaptive daydreaming: A longitudinal analysis. Psychology of Consciousness: Theory, Research, and Practice. https://doi.org/10.1037/cns0000293

77. Margherita, G., Caffieri, A., Mariani, R., Filosa, M., Manari, T., Lenzo, V., Quattropani, M. C., Vegni, E., Borghi, L., Castelnuovo, G., Saita, E., Freda, M. F., Varallo, G., Franceschini, C., & Musetti, A. (2022). Dreaming or daydreaming during COVID-19 lockdown: A comparison between maladaptive and nonmaladaptive daydreamers. Psychology of Consciousness: Theory, Research, and Practice, 10(4), 331–345. https://doi.org/10.1037/cns0000333

76. Conte, G., Arigliani, E., Martinelli, M, Di Nola, S., Chiarotti, F. & Cardona, F. (2022). Daydreaming and psychopathology in adolescence: An exploratory study. Early Intervention in Psychiatry, 17(3), 263-271. https://doi.org/10.1111/ eip.13323

75. Metin, S., Gocmen, B., Metin, B. (2022). Turkish validity and reliability study of maladaptive daydreaming. Psychiatry and Behavioral Sciences, 12(1), 1-6. https://doi.org/10.5455/PBS.20210907111754

74. Soffer-Dudek, N. & Theodor‐Katz, N. (2022). Maladaptive Daydreaming: epidemiological data on a newly identified syndrome. Frontiers in Psychiatry, 13, 871041. https://doi.org/10.3389/fpsyt.2022.871041

73. Mándli, K., McCutcheon, L. & Zsila, Á. (2022). Relationship Status and Celebrity Worship: Exploring Differences in Maladaptive Daydreaming, Engagement, and Body Appreciation across Single and Partnered Women. North American Journal of Psychology 24 (2), 235-256.

72. Theodor‐Katz, N., Somer, E., Hesseg, R. M., & Soffer‐Dudek, N. (2022). Could immersive daydreaming underlie a deficit in attention? The prevalence and characteristics of maladaptive daydreaming in individuals with attention‐deficit/hyperactivity disorder. Journal of Clinical Psychology, 1–20. https://doi.org/10.1002/jclp.2335520

2021

71. Sharma, P. & Mahapatra, A. (2021). Phenomenological Analysis of Maladaptive Daydreaming as A New Form of Behavioral Addiction: A Case Series. Indian Journal of Social Psychiatry 37(3), 280-282. Doi: 10.4103/ijsp.ijsp_227_21

70. Mariani, R., Musetti, A., Di Monte, C., Danskin, K., Franceschini, C., & Christian, C. (2021). Maladaptive Daydreaming in Relation to Linguistic Features and Attachment Style. International Journal of Environmental Research and Public Health, 19(1), 386. https://doi.org/10.3390/ijerph19010386

69. Metin, B., Somer, E., Abu-Rayya, H.M., Schimmenti, A., & Göçmen, B. (2021). Perceived Stress During the COVID-19 Pandemic Mediates the Association Between Self-quarantine Factors and Psychological Characteristics and Elevated Maladaptive Daydreaming. International Journal of Mental Health and Addiction, 21(3), 1570-1582. https://doi.org/10.1007/s11469-021-00678-w

68. Yazhini, C.S. (2021). Statistical analysis of impact on daydreaming. International Journal of Research and Analytical Reviews (IJRAR), 8(3), 543-563.

67. Somer, E., Cardeña, E., Catelan, R.F. Soffer-Dudek., N. (2021). Reality shifting: psychological features of an emergent online daydreaming culture. Current Psychology, 1-13. https://doi.org/10.1007/s12144-021-02439-3.

66. Sándor, A., Bugán, A., Nagy, A., Nagy, N., Tóth-Merza, K., & Molnár, J. (2021). Childhood traumatization and dissociative experiences among maladaptive and normal daydreamers in a Hungarian sample. Current psychology, 1–17. https://doi.org/10.1007/s12144-021-02223-3

65. Brenner, R., Somer, E., & Abu-Rayya, H.M. (2021). Personality Traits and Maladaptive Daydreaming: Fantasy Functions and Themes in a Multi-Country Sample. Personality and Individual Differences, 184, 111194. https://doi.org/10.1016/j.paid.2021.111194.

64. Salomon-Small, G., Somer, E., Harel-Schwarzmann, M. & Soffer-Dudek, N. (2021). Maladaptive Daydreaming and Obsessive-Compulsive Symptoms: A confirmatory and exploratory investigation of shared mechanisms. Journal of Psychiatric Research, 136, 343-350. DOI: 10.1016/j.jpsychires.2021.02.017

63. Costanzo, A., Santoro, G., Russo, S., Cassarà, M.S., Midolo, L.,R., Billieux, J., & Schimmenti, A. (2021). Attached to virtual dreams: The mediating role of maladaptive daydreaming in the relationship between attachment styles and problematic social media use. The Journal of Nervous and Mental Disease, 209(9), 656-664. https://doi.org/10.1097/nmd.0000000000001356

62. Musetti, A., Franceschini, C., Pingani, L., Freda, M.F., Saita, EW., Vegni, E., Zenesini, C., Quattropani, M.C., Lenzo, V., Margherita, G., Lemmo, D., Corsano, P., Borghi, L., Cattivelli, R., Plazzi, G., Castelnuovo, G., Somer, E., Schimmenti A. (2021). Maladaptive daydreaming in an adult Italian population during the COVID-19 lockdown. Frontiers in Psychology, 12, 838. https://doi.org/10.3389/fpsyg.2021.631979

61. Sándor, A., Bugán, A., Nagy, A., Bogdán, L.S., & Molnár, L. (2021). Attachment characteristics and emotion regulation difficulties among maladaptive and normal daydreamers. Current Psychology, 1-18. https://doi.org/10.1007/s12144-021-01546-5

60. Somer, E., Abu-Rayya, H. M., & Brenner, R. (2021). Childhood Trauma and Maladaptive Daydreaming: Fantasy Functions and Themes In A Multi-Country Sample. Journal of trauma & dissociation: the official journal of the International Society for the Study of Dissociation (ISSD), 22(3), 288–303. https://doi.org/10.1080/15299732.2020.1809599

59. Wijaya, R. B. A. (2021). Kondep Diri Pada Masa Dewasa Awal Yang Mengalami Maladaptive Daydreaming. (Self-Concept in Early Adulthood Experiencing Maladaptive Daydreaming). Al-Qalb: Jurnal Psikologi Islam, 12(2), 179-193. (In Indonesian). https://doi.org/10.15548/alqalb.v12i2.2865

58. Sabzban, M; & Safaei, I. (2021). Associating Psychological Symptoms and Worshipping National Taekwondo Champions as Celebrities: The Mediating Role of Maladaptive Daydreaming and Desire for Fame. Sport Psychology Studies, 10(37), 179-206. (In Persian, English abstract). DOI: 10.22089/spsyj.2021.9537.2051

57. Bashir, M.M.I. (2021). Prevalence of maladaptive daydreaming among medical students at the University of Khartoum, Sudan, in 2020–2021. Middle East Current Psychiatry, 28:41, 1-7. https://doi.org/10.1186/s43045-021-00122-8

56. Kammad, Z.A., Al-Sabbagh, A.B., & Hussain, M.A. (2021). Prevalence of Proneness to Maladaptive Daydreaming Syndrome In Basra Medical Students. Elementary Education Online, 20 (1), 2009-2013. doi:10.17051/ilkonline.2021.01.219

55. Caner Yam, F. (2021). Examining the Concept of Maladaptive Daydreaming with the Film Analysis Method. Psikiyatride Guncel Yaklasimlar, 13, 27-39. Doi: 10.18863/pgy.877490 (In Turkish).

54. Yazhini, C.S. (2021). Statistical analysis of impact on daydreaming. International Journal of Research and Analytical Reviews, 8(3), 543-563.

53. Perrotta, G. (2021). The state of consciousness: From perceptual alterations to dissociative forms. Analysis of neurobiological and clinical profiles. Journal of Neurology, Neurological Science and Disorders, 7(1), 6-18. DOI: 10.17352/jnnsd.000042

52. Sharma P. & Mahapatra A. (2021). Phenomenological analysis of maladaptive daydreaming associated with internet gaming addiction: a case report. General Psychiatry, 34(2). e100419. doi:10.1136/ gpsych-2020-100419

51. Wijaya, R. B. A. (2021). Self-Concept in Early Adulthood Experiencing Maladaptive Daydreaming. Al-Qalb: Jurnal Psikologi Islam, 12(2), 179-193. https://doi.org/10.15548/alqalb.v12i2.2865 (In Indonesian, English abstract).

2020

50. Soffer-Dudek, N., Somer, E., Abu-Rayya, H. M., Metin, B., & Schimmenti, A. (2020). Different cultures, similar daydream addiction? An examination of the cross-cultural measurement equivalence of the Maladaptive Daydreaming Scale. Journal of Behavioral Addictions, 9(4), 1056-1067. https://doi.org/10.1556/2006.2020.00080

49. Somer, E., Abu-Rayya, H.M., Schimmenti, A., Metin, B., Brenner, R. Ferrante, E., Göçmen, B., and Marino, A. (2020). Heightened Levels of Maladaptive Daydreaming Are Associated With COVID-19 Lockdown, Pre-existing Psychiatric Diagnoses, and Intensified Psychological Dysfunctions: A Multi-country Study. Frontiers in Psychiatry, 11, 587455. https://doi.org/10.3389/fpsyt.2020.587455

48. Ferrante, E., Marino, A., Guglielmucci, F., & Schimmenti, A. (2020). The Mediating Role of Dissociation and Shame in the Relationship Between Emotional Trauma and Maladaptive Daydreaming. Psychology of Consciousness: Theory, Research, and Practice, 9(1), 27. http://dx.doi.org/10.1037/cns0000253

47. Sándor, A., Münnich, Á., & Molnár, J. (2020). Psychometric properties of the Maladaptive Daydreaming Scale in a sample of Hungarian daydreaming-prone individuals. Journal of Behavioral Addictions, 9(3), 853-862. https://doi.org/10.1556/2006.2020.00050

46. Gysi, J. (2020). Maladaptive Tagträumen, In Diagnostik von Traumafolgestörungen: Multiaxiales Trauma-Dissoziations-Modell nach ICD-11. Bern, Switzerland: Hogrefe, p.271 (in German). https://doi.org/10.1024/86227-000

45. Ross, C.A., Ridgway, J., & George, N. (2020). Maladaptive Daydreaming, Dissociation, and the Dissociative Disorders. Psychiatric Research & Clinical Practice, 2(2), 53-61. https://doi.org/10.1176/appi.prcp.20190050

44. Greene, T., West, M. & Somer, E. (2020). Maladaptive daydreaming and emotional regulation difficulties: A Network Analysis. Psychiatric Research, 285, 112799. https://doi.org/10.1016/j.psychres.2020.112799

43. West, M. & Somer, E. (2020). Empathy, emotion regulation and creativity in immersive daydreaming. Imagination, Cognition and Personality, 39(4), 358-373. DOI: 10.1177/0276236619864277

42. Vázquez-Rivera, S., de la Vega-Rodríguez, I., García-Villamor, M., Díaz-Marsá, M., & Carrasco-Perera, J. L. (2020). Trastorno de Ensoñación Excesiva: Características Clínicas y Neuropsicológicas del PrimerCaso Descrito en España. Revista de Casos Clínicos en Salud Mental, 8(1), 2. Revista De Casos Clínicos En Salud Mental, 8(1).

41. Alenizi, M.M., Alenazi, S.D., Almushir, S., et al. (2020) Impact of Maladaptive Daydreaming on Grade Point Average (GPA) and the Association Between Maladaptive Daydreaming and Generalized Anxiety Disorder (GAD). Cureus, 12(10). https://doi.org/10.7759/cureus.10776

40. Adeniz, E. (2020). Daydreaming Overdose: Maladaptive Daydreaming. PsiNossa, 60, 29-31 (In Turkish)

39. Dujić, G., Antičević, V. & Mišetić, I. (2020). Contribution of Maladaptive Daydreaming to the Level of Psychological Distress and Coping Strategies. Socijalna psihijatrija (Social Psychiatry), 48 (1), 3-19. https://doi.org/10.24869/spsih.2020.3 (In Serbian)

38. Vally, Z., Moussa, D., Khalil, E., Al Fahel, A., Al Azry, N., & Jafar, N. (2020). Celebrity worship in the United Arab Emirates: An examination of its association with problematic Internet use, maladaptive daydreaming, and desire for fame. Psychology of Popular Media, 10(1), 124–134. https://doi.org/10.1037/ppm0000276

2019

37. Gervasi, S., Santoro, G. & Schimmenti, A. (2019). Maladaptive daydreaming: teoria, ricerca e implicazioni cliniche del disturbo da sogni a occhi aperti. Psichiatria & Psicoterapia, 38(4), 237-249. (Maladaptive daydreaming: theory, research, and clinical implications of daydreaming, In Italian).

36. Marcusson-Clavertz, D., West, M., Kjell, Somer, E. (2019) A daily diary study on maladaptive daydreaming, mind wandering, and sleep disturbances: Examining within-person and between-persons relations. PLoS ONE, 14(11): e0225529. https://doi.org/10.1371/journal. pone.0225529

35. Schimmenti, A., Somer, E. & Regis, M. (2019). Maladaptive daydreaming: Towards a nosological definition. Annales Medico-Psychologiques, 177(9), 865-874. https://doi.org/10.1016/j.amp.2019.08.014

34. Ross, C.A., West, M., & Somer, E. (2019). Self-Reported Medication and Recreational Drug Effectiveness In Maladaptive Daydreaming. Journal of Nervous and Mental Disease, 208(1), 77-80. https://doi.org/10.1097/nmd.0000000000001091

33. Wang, Q., Dong, X., & Li, X. (2019). rTMS as an add-on treatment for maladaptive daydreaming over 10 years in patients with schizophrenia: A case report. Asian Journal of Psychiatry, 43, 208–209. https://doi.org/10.1016/j.ajp.2017.10.025

32. Rebello, P., Johnson, K., D'Souza, P., Rao, P.R., & Malamarthi, S. (2019). A case report on maladaptive daydreaming. Galore International Journal of Health Science and Research, 4(1), 33-36.

31. Abu-Rayya, H. M., Somer, E., & Meari-Amir, S. (2019). The Psychometric Properties of the Arabic 16-Item Maladaptive Daydreaming Scale (MDS-16-AR) in a Multicountry Arab Sample. Psychology of Consciousness: Theory, Research, and Practice, 6(2), 171-183. http://dx.doi.org/10.1037/cns0000183

30. Abu-Rayya, H. M., Somer, E., & Knane, H. (2019). Maladaptive Daydreaming Is Associated With Intensified Psychosocial Problems Experienced by Female Survivors of Childhood Sexual Abuse. Violence against women, 26(8), 825–837. https://doi.org/10.1177/1077801219845532

29. Somer, E., Abu-Rayya, H.M. & Nsairy Samaan, Z. (2019). Maladaptive daydreaming among recovering substance use disorder patients: its prevalence and mediation of the relationship between childhood trauma and dissociation. International Journal of Mental Health and Addictions, 17(2), 206-216, https://doi.org/10.1007/s11469-018-0011-9

28. Zsila, Á., Urbán, R., McCutheon, L.E. & Demetrovics, Z. (2019). A path analytic review of the association between psychiatric symptoms and celebrity worship: The mediating role of maladaptive daydreaming and desire for fame. Personality and Individual Differences, 151, 109511. https://doi.org/10.1016/j.paid.2019.109511

27. Schimmenti, A. Sideli, L. La Marca, L., Gori, A. & Terrone, G. (2019): Reliability, Validity, and Factor Structure of the Maladaptive Daydreaming Scale (MDS–16) in an Italian Sample. Journal of Personality Assessment 102(5), 689-701. https://doi.org/10.1080/00223891.2019.1594240

26. Somer, E., Somer, L. & Halpern, N. (2019). Representations of maladaptive daydreaming and the self: A qualitative analysis of drawings. The Arts in Psychotherapy. 63, 102-110. https://doi.org/10.1016/j.aip.2018.12.004

25. Miller, A. (2019). Therapeutic neutrality, ritual abuse, and maladaptive daydreaming. A commentary. Frontiers in the Psychotherapy of Trauma and Dissociation, 3(1), 4–11. https://doi.org/10.46716/ftpd.2019.0018

24. Ross, C.A. (2019). Maladaptive daydreaming and therapeutic neutrality: A rejoinder. Frontiers in the Psychotherapy of Trauma and Dissociation, 3(1), 12–13. https://doi.org/10.46716/ftpd.2019.0019

23. Somer, E. (2019). On dissociative identity disorder and maladaptive daydreaming. Frontiers in the Psychotherapy of Trauma and Dissociation, 3(1), 14–18. https://doi.org/10.46716/ftpd.2019.0020

22. Ross, C.A. (2019). Maladaptive daydreaming and dissociation: Both a continuum and a taxon. A rejoinder. Frontiers in the Psychotherapy of Trauma and Dissociation, 3(1), 19–20. https://doi.org/10.46716/ftpd.2019.0021

2018

21. Pietkiewicz, I.J., Nęcki, S., Bańbura, A, & Tomalski, R. (2018). Maladaptive daydreaming as a new form of behavioral addiction. Journal of Behavioural Addictions, 7(3), 838-843. https://doi.org/10.1556/2006.7.2018.95

20. Zsila, Á., McCutcheon, L.E., & Demetrovics, Z. (2018). The association of celebrity worship with problematic Internet use, maladaptive daydreaming, and desire for fame. Journal of Behavioral Addictions, 7(3), 654–664. https://doi.org/10.1556/2006.7.2018.76

19. Jopp, D. S., Dupuis, M., Somer, E., Hagani, N., & Herscu, O. (2018). Validation of the Hebrew Version of the Maladaptive Daydreaming Scale (MDS-H): Evidence for a Generalizable Measure of Pathological Daydreaming. Psychology of Consciousness: Theory, Research, and Practice, 6(3), 242-261. http://dx.doi.org/10.1037/cns0000162

18. Sándor, A., Molnár, J. (2018). Maladaptív álmodozás. In: Kuritárné, Sz. I., Molnár, J., Nagy, A. (Eds). Trauma-eredetű disszociáció. Budapest: Oriold és Társai, 285-302.

17. Bershtling, O., & Somer, E. (2018). The micro-politics of a new mental condition: Legitimization in maladaptive daydreamers’ discourse. The Qualitative Report, 23(8), 1983-2002. https://doi.org/10.46743/2160-3715/2018.3466.

16. Ross, C.A. (2018). The Potential Relevance of Maladaptive Daydreaming in Treatment of Dissociative Identity Disorder in Persons With Ritual Abuse and Complex Inner Worlds. Frontiers in the Psychotherapy of Trauma and Dissociation, 1(2), 160-173. https://doi.org/10.46716/ftpd.2017.0010.

15. Soffer-Dudek, N. & Somer, E. (2018) Trapped in a Daydream: Daily Elevations in Maladaptive Daydreaming Are Associated With Daily Psychopathological Symptoms. Frontiers in Psychiatry, 9(194). https://doi.org/10.3389/fpsyt.2018.00194

14. Somer, E. (2018). Maladaptive daydreaming: Ontological analysis, treatment rationale: a Pilot case study. Frontiers in the Psychotherapy of Trauma and Dissociation, 2(1), 1–22. https://doi.org/10.46716/ftpd.2017.0006

13. Wei, L. (2018). 非适应性白日梦: 概念、测评及共病情况 (Maladaptive daydreaming: concept, assessment and comorbidity). Journal of Nervous Diseases and Mental Health, 18 (1), 61-65 (In Chinese).

12. Anwar, M., Aqeel, M., & Shuja, K.M. (2018). Linking Social Support, Social Anxiety and Maladaptive Daydreaming: Evidence from University Students of Pakistan. Foundation University Journal of Psychology, 2(2), 141-181. https://doi.org/10.33897/fujp2.16

11. Naguy, A., Alamiri, B., & AlDhaen, M. N. (2018). Methylphenidate for Ego-Syntonic Daydreaming. American Journal of Therapeutics, 25(6), e725–e726. https://doi.org/10.1097/MJT.0000000000000742

2017

10. Somer, E. & Herscu, O. (2017). Childhood trauma, social anxiety, absorption and fantasy dependence: Two potential mediated pathways to maladaptive daydreaming. Journal of Addictive Behaviors, Therapy & Rehabilitation, 6(3), 2-5. https://doi.org/10.4172/2324-9005.1000170.

9. Somer, E., Soffer-Dudek, N., & Ross, C. A. (2017). The comorbidity of daydreaming disorder (Maladaptive Daydreaming). Journal of Nervous and Mental Disease. 205(7), 525-530. http://dx.doi.org/10.1097/NMD.0000000000000685

8. Somer, E., Soffer-Dudek, N., Ross, C. A., & Halpern, N. (2017). Maladaptive daydreaming: Proposed diagnostic criteria and their assessment with a structured clinical interview. Psychology of Consciousness: Theory, Research, and Practice, 4(2), 176-189. http://dx.doi.org/10.1037/cns0000114

2016

7. Somer, E. Somer, L. & Jopp, S.D. (2016). Childhood Antecedents and Maintaining Factors in Maladaptive Daydreaming. Journal of Nervous and Mental Disease, 204(6), 471-478. http://dx.doi.org/10.1097/NMD.0000000000000507

6. Somer, E. Somer, L. & Jopp, S.D. (2016). Parallel Lives: A Phenomenological Study of the Lived Experience of Maladaptive Daydreaming. Journal of Trauma and Dissociation, 17(5), 561-576. http://dx.doi.org/10.1080/15299732.2016.1160463

5. Somer, E., Lehrfeld J., Jopp, D.S., & Bigelsen, J. (2016). Development and Validation of the Maladaptive Daydreaming Scale (MDS). Consciousness and Cognition, 39, 77-91. http://dx.doi.org/10.1016/j.concog.2015.12.001

4. Bigelsen, J., Lehrfeld, J.M., Jopp, D.S. & Somer, E. (2016). Maladaptive daydreaming: Evidence for an under-researched mental health disorder. Consciousness and Cognition, 42, 254-266. http://dx.doi.org/10.1016/j.concog.2016.03.017

2011

3. Bigelsen, J., & Schupak, C. (2011). Compulsive fantasy: Proposed evidence of an under-reported syndrome through a systematic study of 90 self-identified non-normative fantasizers. Consciousness and Cognition, 20(4), 1634-1648. http://dx.doi.org/10.1016/j.concog.2011.08.013

2009

2. Schupak, C. & Rosenthal, J. (2009). Excessive daydreaming: A case history and discussion of mind wandering and high fantasy proneness. Consciousness and Cognition, 18(1), 290-292. http://dx.doi.org/10.1016/j.concog.2008.10.002

2002

1. Somer, E. (2002). Maladaptive daydreaming: A qualitative inquiry. Journal of Contemporary Psychotherapy, 32(2), 195-210. https://doi.org/10.1023/A:1020597026919
